# Enhanced expression of cohesin loading factor NIPBL confers poor prognosis and chemotherapy resistance in non-small cell lung cancer

**DOI:** 10.1186/s12967-015-0503-3

**Published:** 2015-05-12

**Authors:** Weizhen Xu, Yinyin Ying, Lihong Shan, Jianguo Feng, Shengjie Zhang, Yun Gao, Xiaoling Xu, Yinli Yao, Chihong Zhu, Weimin Mao

**Affiliations:** Zhejiang Key Laboratory of Diagnosis and Treatment Technology on Thoracic Oncology (Lung and Esophagus), 38, Guangji Load, Hangzhou, Zhejiang 310022 China; Cancer Research Institute, Zhejiang Cancer Hospital, 38, Guangji Load, Hangzhou, Zhejiang 310022 China; Department of Thoracic Surgery, Zhejiang Cancer Hospital, 38, Guangji Load, Hangzhou, Zhejiang 310022 China

**Keywords:** NIPBL, Cohesin, Non-small cell lung cancer, Prognosis, Chemotherapy resistance

## Abstract

**Background:**

NIPBL, the sister chromatid cohesion 2 (SCC2) human homolog, is a cohesin loading factor which is essential for deposition of cohesin onto the sister chromatid. Recent studies have shown that NIPBL contribute to sister chromatid cohesion and plays a critical role in development, DNA repair, and gene regulation. In this study, we measured the expression of NIPBL in clinical non-small cell lung cancer specimens, and determined its effects on cellular processes and chemosensitivity *in vitro*.

**Methods:**

NIPBL immunohistochemistry was performed on 123 lung adenocarcinoma samples. Through knockdown of NIPBL protein expression, non-small cell lung cancer cell lines were used to test the potential involvement of NIPBL silencing on cell proliferation, migration, invasion, and apoptosis. Chemosensitivity was assessed with clonogenic assays, and chromatin immunoprecipitation assays were performed to analyze the relationship between NIPBL and signal transducers and activators of transcription 3 (STAT3).

**Results:**

Immunohistochemical analysis showed that high expression of NIPBL was strongly correlated with poor prognosis, tumor differentiation, and lymph node metastasis. Survival analysis further indicated that NIPBL expression was a potential prognostic factor for non-small cell lung cancer. Knockdown of NIPBL in non-small cell lung cancer cell lines significantly reduced cellular proliferation, migration, and invasion, and enhanced cellular apoptosis and sensitivity to cisplatin, paclitaxel, and gemcitabine hydrochloride. NIPBL bound to the promoter region of the *STAT3* gene, directly regulating the expression of STAT3.

**Conclusions:**

These data suggested that NIPBL played a significant role in lung carcinogenesis. NIPBL expression conferred poor prognosis and resistance to chemotherapy in non-small cell lung cancer, suggesting that NIPBL may be a novel therapeutic target.

## Background

In eukaryotic cells, a group of conserved proteins termed cohesins form a complex that holds the two sister chromatids together during DNA replication in S phase, until their separation at the onset of anaphase. This biological process is termed sister chromatid cohesion. The core cohesin complex consists of four proteins, SCC1 (also known as RAD21), SCC3 (STAG1, STAG2), SMC1A, and SMC3. These proteins form a ringlike structure which can encircle the DNA [1]. A number of regulatory factors have been found to play roles in cohesin function, such as the SCC2/SCC4 loading complex, the cohesin maintenance WAPL/PDS5 complex proteins, and protein acetyltransferase ESCO2 [2,3].

Cohesin loading factor SCC2 is evolutionarily well conserved [4]. NIPBL is a human homolog of SCC2. NIPBL/Nipped-B protein is found in the nuclei of all eukaryotic cells, where it interacts with the cohesin complex that plays a role in loading cohesin onto chromosomes. In addition to its role in sister chromatid cohesion, NIPBL/Nipped-B/Nipbl also functions in the regulation of gene expression [5,6]. In *Drosophila*, for example, it was shown that Nipped-B was required for long range promoter enhancer activation of the *Cut* and *Utrabithorax* homeobox genes, which control multiple aspects of development [7]. Nipped-B and cohesin colocalized to sites enriched within the promoter regions of the dysregulated genes [8]. Mutations in *NIPBL* have been identified in approximately 60% of individuals with Cornelia de Lange syndrome (CdLS) [9,10]. This dominant, genetically heterogeneous developmental disorder is characterized by craniofacial anomalies, growth retardation, upper limb defects, intellectual disability, and gastrointestinal and genitourinary developmental abnormalities. Observations in CdLS patients and mouse models showed that heterozygous NIPBL mutations produced only a 25 ~ 30% drop in transcript levels [11-13]. Gene expression profiling demonstrated that small changes in NIPBL/Nipbl levels led to significant transcriptional dysregulation of many genes in *Nipbl*+/− mice, and in *NIPBL* mutant human cells, the expression levels of NIPBL/Nipbl correlated with the phenotypic severity of the CdLS disorder [13,14]. These results implied that extreme sensitivity to development correlated with small changes in NIPBL activity. Most recently, a study using different human cell lines and lymphoblastoid cell lines derived from CdLS patients demonstrated that high affinity NIPBL binding sites almost exclusively localized to the promoters of active genes, in a cohesin-independent manner [15]. Further studies found that NIPBL could recruit histone deacetylases, and repress promoter activity [16]. These observations suggested that NIPBL expression levels were critical for cells. NIPBL may act as a transcription factor and may have an upstream role in gene regulation in multiple ways through interactions with the transcriptional machinery.

It was found that a number of NIPBL binding genes were important during development and were known to be dysregulated in cancer. For example, Nipped-B/Nipbl could positively regulate the transcription of *Myc, Oct4*, and *Nanog* genes in *Drosophila* and mouse embryonic stem cells [17,18]. These genes are crucial for cell proliferation and maintenance of pluripotency. In addition, data from the Somatic Mutation in Cancer (COSMIC) database suggested that rare *NIPBL* mutations were identified in lung carcinoma, breast carcinoma, and colorectal tumors [19]. In a genome-wide functional screen, *NIPBL* was identified as one of 11 signature genes whose silencing caused tamoxifen resistance [20]. Although these results and analyses implied that abnormal NIPBL may be a significant feature or might contribute to carcinogenesis, there are no data currently available from clinical cancer samples.

Lung cancer is one of the most common malignancies worldwide. In the past decade, lung adenocarcinoma, a subtype of non-small cell lung cancer (NSCLC), has become the most common histological type among all lung cancers diagnosed. Despite the common occurrence, understanding of the NSCLC genetic molecular background and underlying molecular mechanism leading to its growth and progression remain incomplete. Here, we evaluated the expression of NIPBL in clinical samples of lung adenocarcinoma, and found that almost one-third of the samples expressed high levels of NIPBL protein, and high NIPBL expression was associated with poor clinical outcome. Small interference RNA (siRNA) mediated knockdown of NIPBL significantly impaired cellular proliferation, migration, and invasion, and enhanced the proapoptotic effects of chemotherapy on NSCLC cell lines. These results provided the first evidence that abnormal NIPBL expression might either play a direct role in carcinoma progression or predict therapeutic outcomes of NSCLC.

## Methods

### Patients and samples

Lung adenocarcinoma specimens were obtained from patients who underwent pulmonary lobectomy at Zhejiang Cancer Hospital from 2008 to 2009, and the study was approved by the Ethic Committee of Zhejiang Cancer Hospital and all patients gave informed consent. Patient medical records were review to obtain tumor staging, pathology, and survival information. Clinicopathological classification and staging of these samples were based on the World Health Organization histological classification of tumors of the lung [21] and AJCC Cancer Staging Manual, 7th Edition(2010)_ lung cancer. Patient median age was 57.03 years (range from 29 to 81 years). Among 136 cases of lung adenocarcinoma that we examined, follow-up results were available on 123 cases. Patient median follow-up was 50 months.

### Immunohistochemistry and staining evaluation

Immunohistochemistry was performed on 4 μm sections of paraffin-embedded tissue samples. Immunohistochemical staining was done using a SPlink Detection Kits (SP-9000, ZSGB-BIO, China) according to the manufacture’s protocol. Briefly, de-paraffinized and rehydrated tissue sections were treated for antigen retrieval in 10 mM Tris–HCl (pH 9.0) buffer containing 1 mM EDTA for 10 minutes at 100°C, then treated with 3% H_2_O_2_ for 10 minutes to remove endogenous peroxides, Sections were incubated with anti NIPBL antibody in rabbit (HPA040834, Sigma, CA, USA) at 1:50 dilution, the signal was detected using DAB (3,3′-diaminobenzidine) substrate. Immunohistochemical evaluation was performed separately by two pathologists knowing none about the patients’ clinical characteristics. Nuclear NIPBL expression was assessed for the percentage of positive cells (quantity score:no staining is scored as 0, 1–10% of cells stained scored as 1, 11–50% as 2, 51–80% as 3, and 81–100% as 4), and intensity (staining intensity score: 0 = negative; 1 = weak; 2 = moderate, and 3 = strong). An overall protein expression score was calculated by multiplying the quantity and staining intensity scores. The overall scores could range from 0 to 12. For statistical analysis, an overall staining score of ≥6 was considered to be high expression of NIPBL protein, score of 0–5 was considered as low expression.

### Cell lines and transfection

The human NSCLC cell lines H1299 and A549 were obtained from the American Type Culture Collection (ATCC, Rockville, MD, USA). H661, H1650 (lung adenocarcinoma cell line) were obtained From Cell Bank at the Chinese Academy of Sciences (Shanghai, China). H1299, H661 and H1650 cells were grown and maintained in RPMI 1640 medium supplemented with 10% fetal bovine serum (FBS) and 1% penicillin/streptomycin. A549 cells were cultured in F-12 Medium supplemented with 10% fetal bovine serum.

Multiple siRNAs against human NIPBL and a scramble control siRNA were constructed by Genepharma (Genepharma, Shanghai, China). Two siRNAs (siNIPBL-N2 and siNIPBL-N3) with the best silencing potential (estimated using Western blot analysis, date not shown) were chosen to silence NIPBL expression (The sequence for NIPBL siRNA: siNIPBL-N2: 5′-GCUCGGAACAAAGCAAUUA-3′, siNIPBL-N3:5′-GCGGCAAUGUAUGAUAUAATT-3′. The sequence for control siRNA: siNIPBL-NC: 5′-GGUUGCCGACUCGUUAAUATT-3′). Thansfection with siRNA was performed using Oligofectamine (Invitrogen, Carlsbad, CA), all procedures were performed according to manufacturer’s instructions.

### Cell viability assay

The cell viability determined by MTT (Sigma-Aldrich, St. Louis, MO) assay. 24 h after transfected with siRNA, cells were trypsinized and plated in quintuplicates in 96-well plates at the density of 1.5 × 10^3^ per well (H1299) or 2.0 × 10^3^ per well (H1650), respectively. The cells were cultured for 4 h (day0, baseline) or 2–6 day. For colorimetric analysis, the absorbance value (OD) at 490 nm was measured with Multiskan Spectrum UV/visible Mocroplate Reader (Thermo Labsystems, MA, USA). The ratio of the absorbance relative to baseline was calculated.

### Clonogenic survival assay

Cell survival was measured based on colony formation. H1299 and H1650 cells were transfected with siRNA respectively. 24 h after transfection, cells (500cells/well) seeded in 12-well plates. After incubation for 14 days, cells were fixed and stained with Crystal Violet to detect colonies. Colonies containing at least 50 cells were counted under microscope. The experiment was performed in triplicate.

Two day after siRNA transfection, H1299 cells were treated with various concentrations of cisplatin, Paclitaxel and Gemcitabine Hydrochlorid for 24 h, then cells (500cells/well) were diluted in complete medium and seeded in 12-well plates. Clonogenic survival assays was performed as the same as above.

### Cell migration and invasion assays

Cell migration assays were performed using transwell from Costar with 6.5 mm diameter and 8.0 μm pore size, added 1 × 10^4^ cells in 300 μl serum-free media into upper chamber of transwell, assessed the migration potency after 24 h’s incubation. Count the number of cells that migrated across the filter in 4 randomly high-power fields per well. Three identical replicates preformed.

For cell invasion assays, 40 μl of the diluted matrigel was added into the upper chamber of transwell, 5 × 10^4^ cells in serum-free media were added to the upper chamber, after incubation for 24 h, count the number of migrated cells in 4 randomly high-power fields per well. All experiments were performed in triplicate.

### Flow cytometry analysis of apoptosis

48 h after transfection, H1299 cells were treated with lower concentration of cisplatin (0.5 ng/ml), Gemcitabine hydrochlorid (0.02 ng/ml) or paclitaxel (0.2 ng/ml) for 24 h. The Alexa Fluor 488 Annexin V/Dead Cell Apoptosis Kit (Invitrogen, Oregon, USA) was used for apoptosis analysis. Cells were collected and resuspended in staining solution, the staining cells (1 × 10^5^) were analysis with a flow cytometer (BD FACSCalibur, CA, USA).

### Quantitative real time PCR analysis

Total RNA was extracted from cultured cells by using Trizol reagent (Invitrogen, USA) according to the manufacturer’s instruction. cDNA synthesis using a PrimeScript ™ RT Reagent Kit (Takara, Dalian, China). Real time PCR was performed using Applied Biosystems 7500 real-time PCR System (ABI, CA, USA) to measure mRNA levels of *NIPBL, STAT3, Mcl-1, c-Myc*, *β-actin* was amplified as an internal control. The relative expressions of above genes were determined from three independent experiments. Primers used were listed in Table 1.

**Table 1:** **Primers for quantitative real-time PCR**

	Primer
*NIPBL*-F	AGCAGAGACCTGATGGGCGA
*NIPBL*-R	TGTCGCTCTGATTCACCCCTG
*STAT3*-F	TGACGGAGAAGCAGCAGATG
*STAT3*-R	TCCTGGAGATTCTCTACCACTT
*Mcl-1*-F	GAGACCTTACGACGGGTTGG
*Mcl-1*-R	GAGAGTCACAATCCTGCCCC
*c-Myc*-F	CGGGTAGTGGAAAACCAGCA
*c-Myc*-R	AGAAATACGGCTGCACCGAG
*Beta actin*-F	TGGCACCCAGCACAATGAA
*Beta actin*-R	CTAAGTCATAGTCCGCCTAGAAGCA

### Semi-quantitative Western blot analysis

Total protein was isolated from culture cells according to a previous study [16]. Protein samples (50 μg) were then separated by SDS-PAGE and transferred onto a PVDF membrane (Millipore, Bedford, MA). Primary antibodies use anti-NIPBL antibody (1:1000, ABSea, China), anti-C-Myc antibody, anti-STAT3 antibody, anti-Mcl-1 antibody, anti-Bcl-2 antibody, anti-c-PARP antibody, anti-c-Caspase-3 antibody and anti-Survivin antibody (1:1000, Cell signaling, USA) respectively, Anti-alpha Tubulin antibody (1:1000, Abcam, UK) as the loading control. The relative level of protein expression was quantified by image analyzing software Quantity One (Version 4.5.2) (Bio-Rad, USA) and expressed as means ± SD (n = 3).

### Chromatin immunoprecipitation (ChIP) assays

ChIP assays were preformed in H1299 cells according to the manufacturer’s recommendation (EZ-ChIP™, Millipore, 17–371, USA). Proteins were cross-linked to chromatin by adding formaldehyde to 1% final concentration. Chromatin was incubated with anti-NIPBL antibody (KT55, ABSea, China) overnight at 4°C. DNA was purified using spin columns. Primers used for *STAT3* gene promoter were follows: *STAT3*-p1 (148 bp, 5′-GGAGTACGGGGTTAAATCCACTACC-3′ and 5′-GGACAACAAAAAGAACATGGGTGAC-3′) and *STAT3*-p2 (116 bp, 5′-CTCCTAGCTGCTCTCCTCAT-3′ and 5′-CACGCCGTCATGCATAATTC-3′), PCR products were subjected to electrophoresis using 1% agarose gel.

### Statistical analysis

Data were analyzed using the Statistical Package for the Social Sciences (SPSS), Version 18 (SPSS, Chicago, IL, USA). The Pearson’s chi-squared test was used to analyze the relationship between NIPBL expression and clinicopathologic characteristics. Kaplan-Meier plots were constructed to present the survival outcomes and were compared using a log-rank test. One-way ANOVA, Student’s *t*-test, or chi-square tests were used to compare differences between groups. A two-tailed *P*-value test was used in all analyses, and *P* < 0.05 in all cases was considered statistically significant.

## Results

### NIPBL protein expression in lung adenocarcinoma tissues

**Figure 1:**
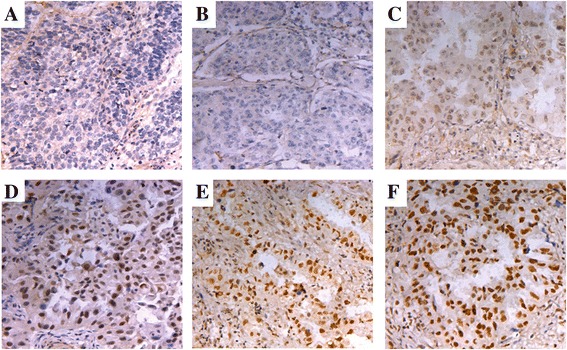
Immunohistochemical staining of NIPBL in lung adenocarcinoma. The overall immunohistochemical staining scores could range from 0 to 12. An overall staining score of ≥6 was considered to be high expression of NIPBL protein, score of 0–5 was considered as low expression. Low level of expression of NIPBL in lung adenocarcinoma sample: **(A)** Absence of nuclear NIPBL expression, overall staining score is 0, **(B)** overall staining score is 1–2. High level expression of NIPBL in lung adenocarcinoma samples: **(C)** overall staining score is 6, **(D)** overall staining score is 8, **(E)** and **(F)** overall staining score are 12. Original magnification: ×200.

NIPBL protein was localized mainly in the nucleus, and no staining or minimal weak staining was present in the cytoplasm in all cases. Expression of NIPBL in lung adenocarcinoma ranged from weak to strong as determined by staining (Figure 1). According to our immunohistochemical staining evaluation, almost one-third of the lung adenocarcinoma samples expressed high levels of NIPBL protein (39/123 cases, 31.7%).

### Association between NIPBL expression and clinicopathological features in lung adenocarcinoma

**Figure 2:**
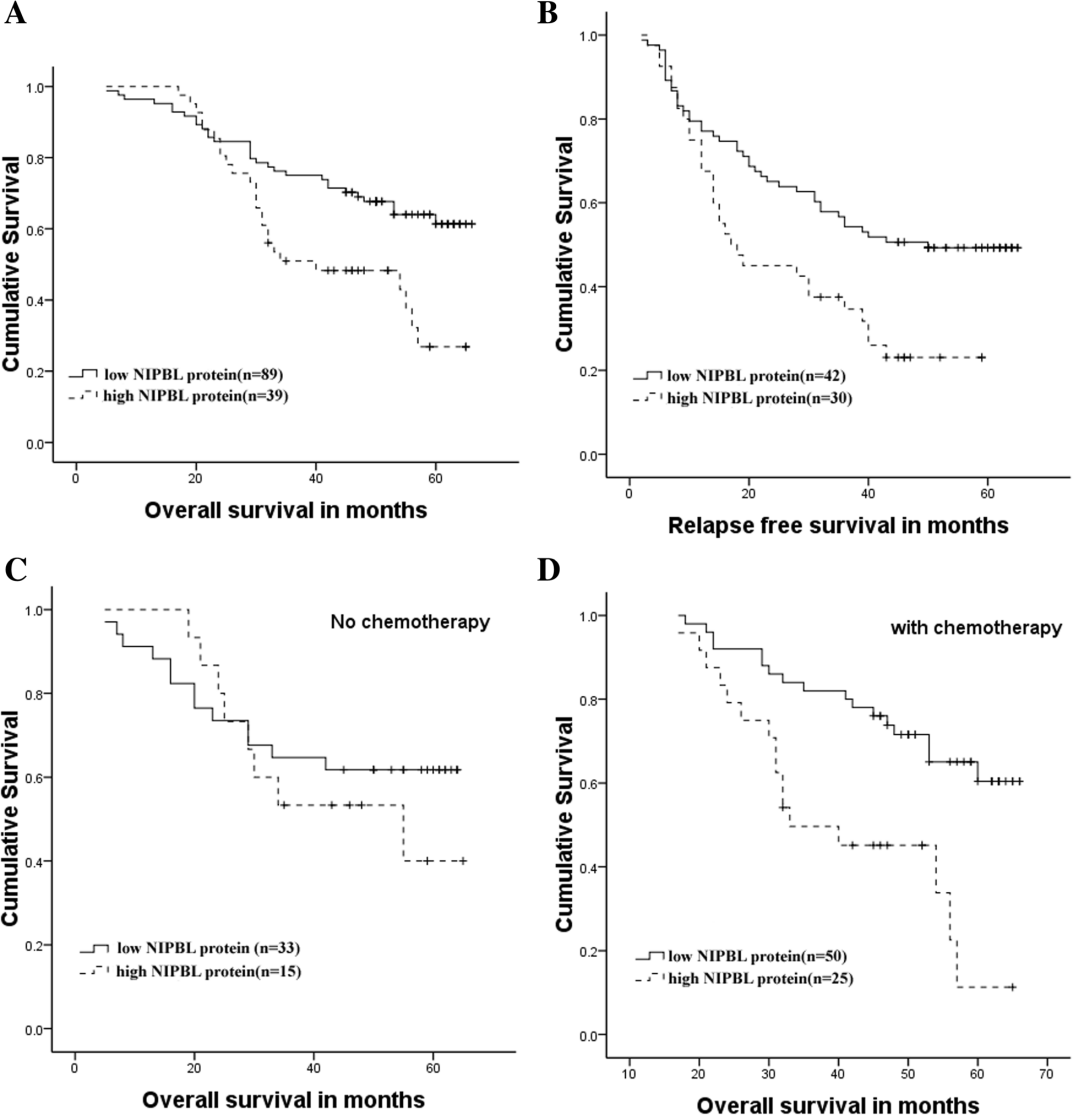
Overall survival and relapse-free survival analysis in lung adenocarcinoma patients according to NIPBL expression. **(A)** Kaplan-Meier analysis of tumor-specific overall survival by NIPBL expression in all lung adenocarcinoma patients (*P* =0.003) (n = 123). **(B)** Kaplan-Meier analysis of relapse free survival by NIPBL expression (*P* =0.006) (n = 72). **(C)** Overall survival in patients without chemotherapy (*P* =0.393) (n = 48), **(D)** Overall survival in patients treated with chemotherapy by NIPBL expression (*P* =0.001) (n = 75).

As shown in Table 2, we found that NIPBL expression was positively correlated with tumor differentiation (*P* = 0.003) and lymph node metastasis (*P* = 0.029). No significant associations were found between NIPBL expression and age, gender, or tumor, node, and metastasis (TNM) stage (*P* > 0.05). High expression of NIPBL was associated with poor differentiation and a poor prognosis. Using the Kaplan-Meier analysis method, we found that there was a significant correlation between high NIPBL expression and shorter median overall survival (*P* = 0.003) (Figure 2A). Moreover, the relapse free survival was significantly higher in the low NIPBL level group than in the high NIPBL level group (*P* = 0.006) (Figure 2B). In addition, there was no correlation between NIPBL expression and overall survival among patients who were not treated with chemotherapy (*P* = 0.393) (Figure 2C), whereas among patients treated with chemotherapy, there was a significantly shorter overall survival in patients whose tumors were positive for NIPBL expression (*P* = 0.001) (Figure 2D).

**Table 2:** **Association of NIPBL expression with clinical characteristics of 123 patients with lung adenocarcinoma**

**characteristics**	**Patients (n = 123), n(%)**	**NIPBL expression**		***P***
		**Low, n (%)**	**High, n (%)**	
**Gender**				
Male	81 (65.9)	52 (61.9)	29 (74.4)	0.175
Female	42 (34.1)	32 (38.1)	10 (25.6)	
**Age (years)**				
<50	37 (30.1)	26 (31.0)	11 (28.2)	0.757
≥50	86 (69.9)	58 (69.0)	28 (71.8)	
**Tumor differentiation**				
Well differentiated	34 (27.6)	29 (34.5)	5 (12.8)	0.003
Moderately differentiated	47 (38.2)	34 (40.5)	13 (33.4)	
Poorly differentiated	42 (34.2)	21 (25.0)	21 (53.8)	
**Lymph node metastasis**				
Positive	81 (65.9)	50 (59.5)	31 (79.5)	0.029
Negative	42 (34.1)	34 (40.5)	8 (20.5)	
**Disease stage**				
I	36 (29.3)	26 (31.0)	10 (25.6)	0.794
II	53 (43.1)	36 (42.9)	17 (43.6)	
III	34 (27.6)	22 (26.1)	12 (30.8)

### NIPBL expression in NSCLC cell lines

**Figure 3:**
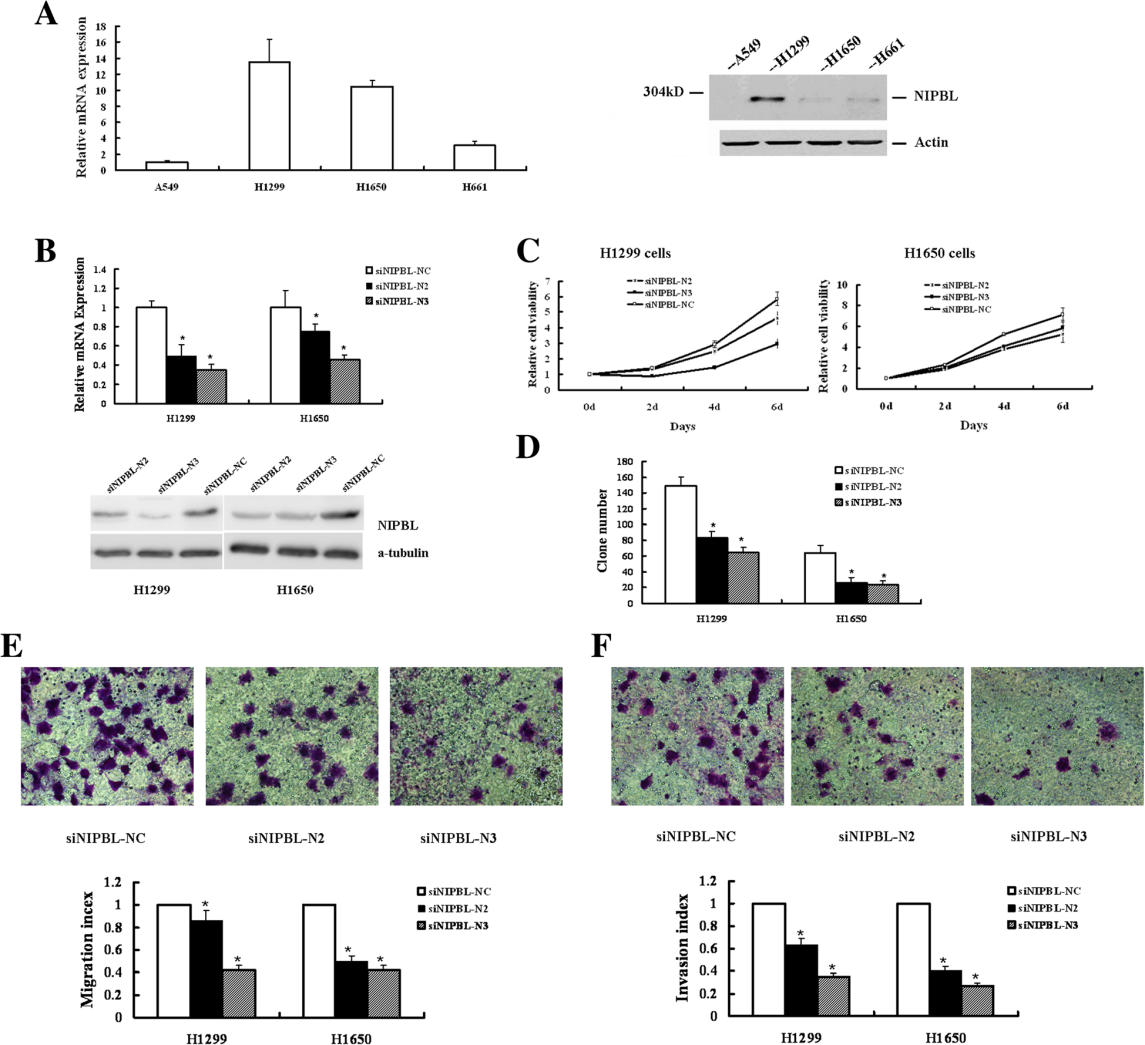
Knockdown of NIPBL inhibited proliferation, migration, and invasion in H1299 and H1650 human NSCLC cell lines. **(A)** RT-PCR analysis of NIPBL mRNA level and Western blot analysis of NIPBL protein level in four human NSCLC cell lines. **(B)** Relative levels of NIPBL mRNA and protein expression in H1299 and H1650 lung cancer cells transfected with siNIPBL-N2, −N3 or –NC (non-targeting scramble control siRNA). Based on morphometric analysis of immunoblot data, in H1299 cell line, siRNA-N2 caused 47 ± 6% reduction, and siRNA-N3 caused 29 ± 4% reduction in NIPBL protein level, in H1650 cell line, siRNA-N2 caused 41 ± 4% reduction, and siRNA-N3 caused 43 ± 7% reduction in NIPBL protein level. **(C)** *In vitro* growth curves of H1299 and H1650 NSCLC cells which transfect with siNIPBL-N2,-N3, and –NC for 0–6 days. Cell viability was determined by MTT assay. For colorimetric analysis, the absorbance value (OD) at 490 nm was measured in the cells which cultured for 4 h, as the baseline of day0. Data represented mean ± SD for three replicate experiments. **(D)** Clone formation assays of NIPBL knockdown H1299 cells and H1650 cells. Cells were seeded at the cell numbers indicated, stained with crystal violet after 2 weeks’ culture. Experiments were performed in triplicates. **(E)** The *in vitro* migratory ability of NIPBL downregulated H1299 and H1650 cells was determined by *in vitro* migration. Cells migrated through the memberane were viewed at × 400 magnifications under light microscope, counted in 4 independent visual fileds SEMper transwell membrane. Cell numbers were presented as values of means ± SD of triplicate experiments. * *P* <0.05. **(F)** The *in vitro* invasive ability of NIPBL downregulated H1299 and H1650 cells was further determined by same assay, as described in **(E)**.

Variations in NIPBL protein and mRNA expression levels in four lung cancer cell lines H1299, A549, H661, and H1650 were quantitated by using the quantitative reverse-transcription polymerase chain reaction (qRT-PCR) and western blot analysis. All four lung cancer lines expressed the NIPBL protein, especially the H1299 and H1650 cell lines, in which NIPBL protein expression was high (Figure 3A).

### Knockdown of NIPBL inhibited proliferation, migration, and invasion in H1299 and H1650 human NSCLC cell lines

We used two efficient siRNAs (siNIPBL-N2 and siNIPBL-N3) to downregulate the NIPBL expression in H1299 and H1650 cell lines. Examination of NIPBL mRNA and protein expression showed a statistical reduction in both NSCLC cell lines transfected with siNIPBL-N2 and siNIPBL-N3, compared to the control cells which were transfected with siNIPBL-NC, a control siRNA. In H1299 cell line, relative to the control siRNA-NC trahsfected cells, the relative levels of NIPBL protein were 47 ± 6% for siRNA-N2 transfected cells, 29 ± 4% for siRNA-N3 transfected cells. In H1650 cell line, the relative levels of NIPBL protein were 41 ± 4% for siRNA-N2 transfected cells, 43 ± 7% for siRNA-N3 transfected cells, relative to the control siRNA-NC transfected cells (Figure 3B).

To test the potential involvement of NIPBL silencing on cell growth and proliferation in NSCLC cells, we performed MTT assays and clonogenic survival assays. After 6 days in culture, H1299 cells transfected with siNIPBL-N2 and siNIPBL-N3 showed 78.8% and 50.7% survival, respectively, and H1650 NIPBL knockdown cells showed 81.9% and 73.8% survival, compared to control cells respectively, in a manner that directly correlated with the level of NIPBL expression (Figure 3C). Consistently, we noted that NIPBL silenced cells exhibited a significantly decreased ability to form colonies compared to control cells at 14 days (Figure 3D).

To confirm the functional significance of our clinicopathological results that NIPBL expression was associated with tumor metastasis, we determined the effect of NIPBL downregulation on cell migration and invasion in H1299 and H1650 cells. As shown in Figure 3, treatment with siNIPBL-N2 and siNIPBL-N3 significantly inhibited both H1299 and H1650 cell migration across Transwell® membranes in comparison with control cells (Figure 3E). Similarly, at 24 h, silencing NIPBL expression in the H1299 and H1650 cell lines also significantly decreased the ability of the cells to invade through a Matrigel™ compared with scrambled control siRNA treated cells (Figure 3F).

### Knockdown of NIPBL enhanced sensitivity to chemotherapy drugs in the H1299 cell line

**Figure 4:**
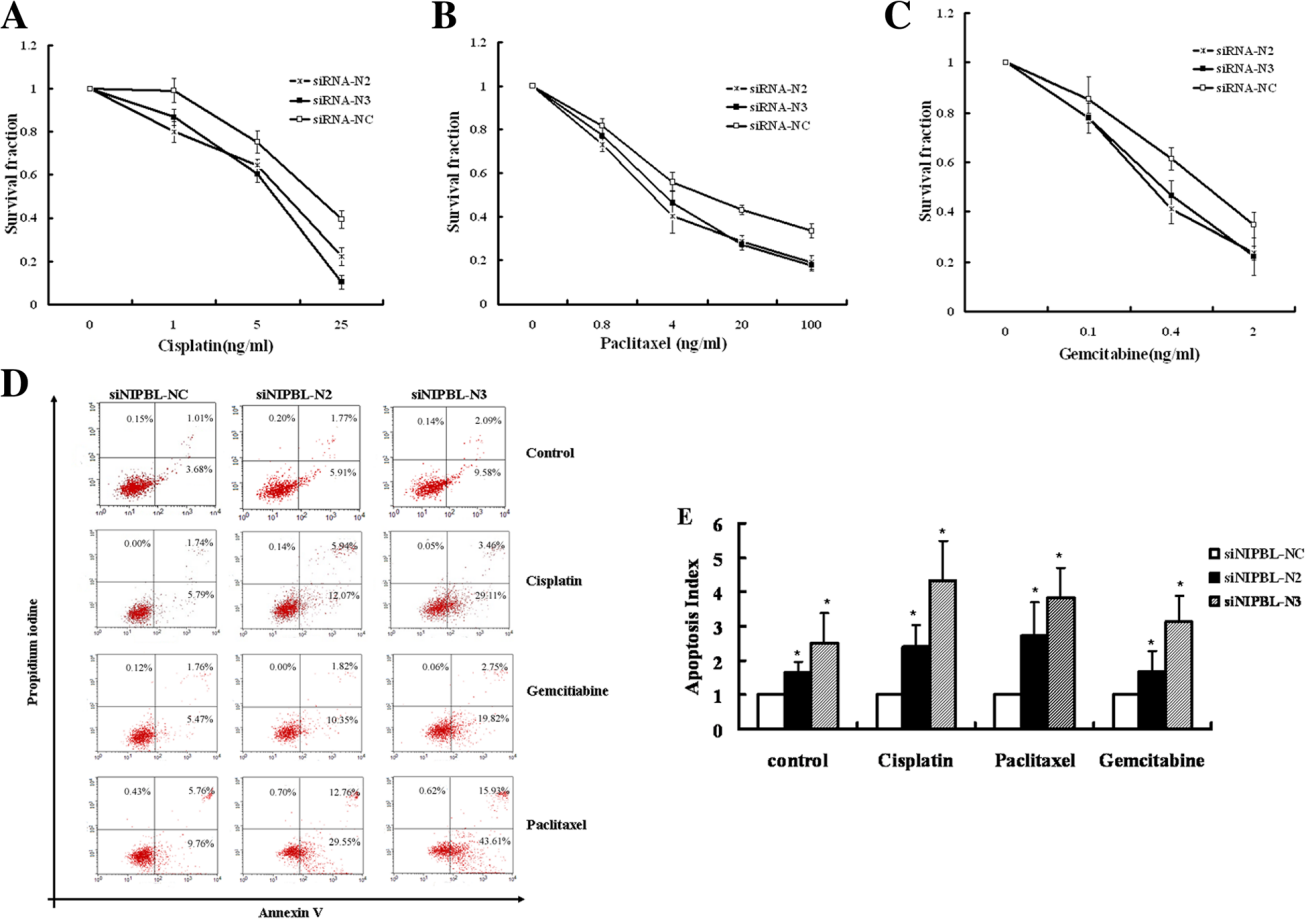
NIPBL knockdown enhanced cellular sensitivity to chemotherapy drugs, and increased chemotherapy drug-induced apoptosis in the H1299 cell line. H1299 cells were transfected with siNIPBL or non-targeting scramble siRNA for 24 h, and then treated with different concentrations of cisplatin, Gemcitabine hydrochlorid and paclitaxel for 24 h, seeded at the cell numbers indicated, cultured for 2 weeks. Clonogenic survival following treatment with: **(A)** cisplatin; **(B)** paclitaxel; **(C)** Gemcitabine hydrochlorid. Data represented mean ± SD for three replicate experiments. **(D)** H1299 cells were transfected with either siNIPBL or non-targeting scramble siRNA as indicated. After 48 h, cells were treated with lower concentration of cisplatin (0.5 ng/ml), Gemcitabine hydrochlorid (0.02 ng/ml) and paclitaxel (0.2 ng/ml) for 24 h. The cells were collected and stained with Annexin-V-FITC and PI. Shown were representative images of three independent experiments, values are expressed as a percentage of Annexin V positive versus total cells. **(E)** Apoptosis index were presented as mean ± SD of triplicate experiments. * *P* <0.05.

To further test the functional significance of our cancer therapy results that NIPBL expression affected sensitivity to chemotherapeutic drug responses, we investigated the effect of downregulating endogenous NIPBL expression on cell growth after exposure to chemotherapeutic drugs. Clonogenic assays were performed on cells that were initially transfected with NIPBL siRNA, and subsequently exposed to varying concentrations of cisplatin, paclitaxel, and gemcitabine hydrochloride. After 14 days, compared to the control cells, both NIPBL knockdown cell cultures showed significant reduction in the clonogenic survival fraction when they were exposed to cisplatin (Figure 4A). Similarly, enhanced sensitivity was observed in the two NIPBL knockdown cell cultures treated with paclitaxel and gemcitabine hydrochloride (Figure 4B, C).

### Knockdown of NIPBL increased apoptosis in the H1299 cell line

To examine whether increased sensitivity to chemotherapeutic drugs was due to an increased apoptotic cell death, the cellular apoptosis ratio of NIPBL knockdown cells treated with lower concentrations of cisplatin, gemcitabine hydrochloride, or paclitaxel was determined. As shown in Figure 4D–E, compared with control cells, silencing of NIPBL increased cell apoptosis significantly after treatment with chemotherapeutic drugs (*P* < 0.05).

### Knockdown of NIPBL inhibited expression of STAT3 and STAT3 target genes, and NIPBL directly regulated the expression of STAT3

**Figure 5:**
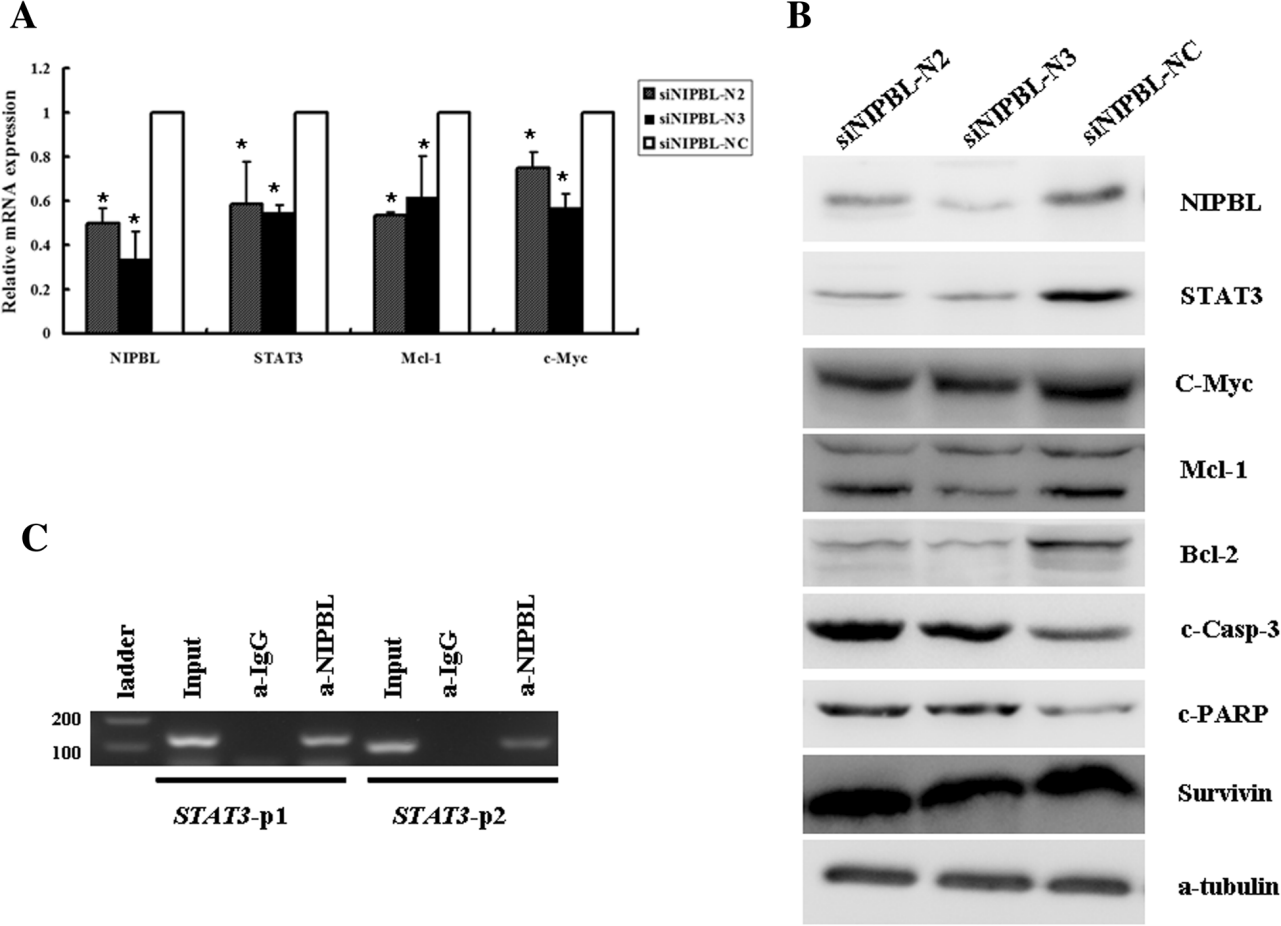
Knockdown of NIPBL inhibited expression of STAT3-related gene, NIPBL directly regulate the expression of STAT3. **(A)** H1299 cells were transfected with siNIPBL or non-targeting scramble siRNA for 48 h, RT-PCR analysis of *STAT3, c-Myc,* and *Mcl-1* mRNA levels. * *P* <0.05. **(B)** After transfection 72 h, H1299 cells collected and subjected to western blot analysis for detection of NIPBL, STAT3-related proteins and apoptosis-associated proteins levels, a-tubulin was used as a loading control. Shown were representative blots of three independent experiments. **(C)** Chromatin-immunoprecipitation (ChIP) assays were used to demonstrate that NIPBL bind to *STAT3* promoter region. Two pair of primer (STAT3-p1 and STAT3-p2) was designed to amplify different fragments of *STAT3* promoter region. Input DNA before immunoprecipitation was used as positive control, as negative control, a nonspecific antibody (a-IgG) was used for precipitation.

Based on the above results, to further explore the mechanisms responsible for modulation of lung cancer cell survival by NIPBL, we performed RNA-seq assays after knockdown of NIPBL protein in H1299 cells (data not shown). We observed that silencing of NIPBL was associated with significantly decreased mRNA expression for *STAT3* and its target genes *c-Myc*, and *Mcl-1*. As illustrated in Figure 5A, qRT-PCR confirmed that the mRNA levels of STAT3 (signal transducers and activators of transcription 3) signaling pathway-related genes, such as *STAT3*, *Mcl-1*, and *c-Myc*, were significantly decreased when NIPBL was knocked down (*P* < 0.05). Importantly, we also confirmed, by western blotting, that knockdown of NIPBL significantly decreased the protein levels of STAT3, Mcl-1, Bcl-2, and c-Myc (Figure 5B). Furthermore, NIPBL knockdown cells also exhibited markedly increased activation of cleaved caspase-3 and cleaved poly ADP-ribose polymerase (PARP), a substrate of caspase-3. However, survivin, an inhibitor of cell death, was not decreased (Figure 5B). Given that the STAT3-related pathway is constitutively activated in diverse cancers, and acts as a potent pro-survival and anti-apoptotic signaling protein, these data demonstrated that NIPBL silencing inhibited cell growth, possibly via downregulating STAT3 and STAT3 target genes in NSCLC cells.

To further analyze the relationship between NIPBL and STAT3, we performed ChIP assays, using anti-NIPBL antibody, followed by PCR with the primers specific for the *STAT3* promoter, as shown in Figure 5C. We found that NIPBL bound to the *STAT3* promoter region, which suggested that NIPBL might directly regulate the expression of STAT3 protein.

## Discussion

In this study, we did a comprehensive analysis of a cohesin loading factor, NIPBL, in NSCLC. The immunohistochemical results demonstrated that increased NIPBL expression was positively associated with tumor differentiation and tumor metastasis, and that high NIPBL expression was associated with shorter overall and relapse free survival in patients with lung adenocarcinoma, the most common type of NSCLC. Our analyses provided compelling evidence that NIPBL expression was a novel prognostic marker in NSCLC, although further studies using a larger independent patient set would be necessary to confirm this observation. The data from cell lines revealed that downregulation of NIPBL strongly correlated with decreased cellular proliferation and viability, impaired migration, impaired invasion ability, and enhanced proapoptotic effects in human NSCLC cells. These findings supported the hypothesis that NIPBL may play a positive role in the carcinogenesis of NSCLC.

Affected CdLS individuals were shown to have altered developmental gene expression and development was reduced by approximately 30% NIPBL transcript levels, but no obvious cohesin-dependent chromosome cohesion defects were observed, suggesting that pathogenesis of CdLS was more likely due to the dysregulation of numerous developmental genes that resulted from mutations in NIPBL [22]. A non-canonical role for NIPBL as a key regulator of gene expression has been proposed [5,23]. In different species, SCC2 has been shown to directly facilitate expression of *myc,* a critical regulator of cell proliferation and protein synthesis [17,22,24]. In mouse embryonic stem cells, Nipbl could promote expression of genes required for pluripotency, such as, *Oct4* and *Nanog* [18]. Numerous proliferation and pluripotency associated genes could be upregulated by NIPBL, suggesting that NIPBL participated in the cellular decision to proliferate or differentiate. *NIPBL* might also act as an oncogene. Our results suggested the overexpression of NIPBL in lung cancer with proliferative potential and aberrant differentiation. These results suggested that increased NIPBL contributed to cancer progression.

Rare NIPBL mutations were also identified in lung carcinoma, breast carcinoma, and colorectal tumors [19], but the pathological importance of these mutations in cancer remains unknown. Additional evidence showed that somatic mutations in the *NIPBL* gene were found in gastric and colorectal cancers with high microsatellite instability [25]. These results suggested the possibility that underexpression of NIPBL was also associated with carcinogenesis. Accurate and timely segregation of sister chromatids in the cell cycle is essential for maintaining genome integrity [26,27]. A possible explanation for underexpression of NIPBL also associated with carcinogenesis was that insufficient NIPBL might interfere with its interaction with the cohesin complex, which leads to genome instability by disturbing the segregation and cohesion of sister chromatids, thus contributing to aneuploidy in carcinogenesis. Therefore, we hypothesized that NIPBL might act as a gatekeeper of cell fate, maintaining the balance and modulation of critical cellular processes through its multiple roles. The adverse outcomes of varying the expression of NIPBL could be due to the different roles of NIPBL in cellular regulation.

Both cisplatin-based and paclitaxel-based chemotherapies are first-line treatments in NSCLC, Gemcitabine hydrochloride is also used in combination with other chemotherapy drugs to treat NSCLC, but their use has been limited by drug resistance. In this study, we found that decreased NIPBL increased apoptosis induced by these chemotherapeutic drugs, and this correlated with the levels of NIPBL expression. The pharmacological activities of these chemotherapeutic drugs are totally different, but their final mechanism of action is activation of apoptosis in cancer cells. It was known that cancer cells developed resistance to cytotoxic agents and this was related to resistance to apoptosis [28]. Therefore, our results supported the concept that NIPBL acted as an apoptosis inhibitor, thus promoting chemotherapeutic drugs resistance. Suppression of NIPBL expression could therefore enhance the cytotoxicity of chemotherapeutic drugs.

By measuring the mRNA and protein levels of components of the cohesin complex in NIPBL knockdown H1299 cell lines, we noted that cohesin core components RAD21 and SMC1A showed similar downregulation that was directly correlated with the level of NIPBL expression (data not shown). Interestingly, altered RAD21 or SMC1A expression was also reported as associated with distinct cancer phenotypes [29-32], although the complete mechanism of cohesin biology remains to be determined. Cohesin core components and their regulatory proteins could function together, or separate cohesin subunits could play different roles in different physiological and pathological contexts. It will be interesting to study how a single protein complex can be involved in so many cellular processes.

Knowledge of how NIPBL regulates molecular pathways is relatively limited. To investigate whether downregulation of NIPBL leads to alterations in transcription, we performed RNA-seq assays after knockdown of NIPBL protein in H1299 cells (data not show). We confirmed that insufficient NIPBL was associated with significant decrease in mRNA and protein expression of STAT3 and its target factors c-Myc, Mcl-1, and Bcl-2. STAT3 is a component of the signal transducers and activators of the transcription (STATs) family, which acts as an important transcription factor, and plays a role in normal development and cancer progression by regulating cell survival, proliferation, and apoptosis [33,34]. STAT3 was frequently constitutively activated in many human cancers, and STAT3 activation was also associated with therapeutic drug resistance [35]. Tumorigenic STAT3 is likely due to the aberrant activity of STAT3’s upstream signaling pathways, such as, JAK2, EGFR, Src and HER2 [36]. C-Myc protein is a key regulator in cell growth and proliferation [37,38], and Mcl-1 protein belongs to the Bcl-2 family which maintains cell survival by inhibiting cell apoptosis [39]. Overexpression of both proteins enhanced many cancer types [38,40,41]. In this study, we showed that NIPBL could directly bind to the promoter region of the *STAT3* gene, suggesting that NIPBL might act as an upstream regulator of STAT3. It is hypothesized that downregulation of NIPBL could inhibit the activation of STAT3, reduce the expression of known STAT3 downstream genes, and contribute to the inhibition of cell growth, migration, invasion, and the induction of apoptosis. In addition, from flies to humans, it is remarkable that positive regulation of c-Myc transcription by Nipped-B/Nipbl/NIPBL is directly and evolutionarily conserved [14,24,42].

Base on previously reports, NIPBL may be involved in gene regulation through various mechanisms. In different model organisms, genome-wide mapping identified extensive colocalization between cohesin and CCCTC-binding factor (CTCF functions as a transcriptional insulator) [8,43-45], but in mouse embryonic stem cells, Nipbl, cohesin and mediator (transcriptional co-activator) colocalized at many sites other than CTCF binding sites [18]. These results suggested that cohesin or Nipped-B/Nipbl regulate gene expression might occur via both CTCF-dependent and CTCF-independent pathways. Recently, NIPBL ChIP analyses have identified high affinity NIPBL binding sites in different cell lines which did not overlap with cohesin binding sites, but overlapped almost exclusively with the promoters of active genes. These results suggested a cohesin-independent role for NIPBL in transcriptional regulation [15]. NIPBL may be involved in gene regulation through various mechanisms in various cell types. Therefore, it will be interesting to elucidate the mechanisms by which NIPBL both negatively and positively regulates the transcription of certain genes in NSCLC.

## Conclusions

In summary, to our knowledge this study was the first to show that NIPBL expression levels could predict the clinical outcome and the resistance to chemotherapy in NSCLC. NIPBL inhibition had *in vitro* effects on proliferation, invasion, and apoptosis control. In the future, precise molecular details of regulation remain to be further studied, and targeting NIPBL might provide a novel therapy for NSCLC.

## Abbreviations

NIPBL: Nipped-B homolog (human)
NSCLC: Non-small cell lung cancer
STAT3: Signal transducers and activators of transcription 3
SMC1: Structural maintenance of chromosome protein 1
SCC3: Sister-chromatide cohesion protein 3
COSMIC: Data from Somatic Mutation in Cancer
siRNA: small interference RNA
ChIP assays: Chromatin immunoprecipitation assay
CTCF: CCCTC-binding factor
PARP: Poly ADP-ribose polymerase
